# Supplementary material for: Splice-Junction-Based Mapping of Alternative Isoforms in the Human Proteome
Source: Cell Rep. Author manuscript; Available in PMC 2020 Jan 15. (PMC6961840; doi:10.1016/j.celrep.2019.11.026)
Supplement: 2 [file NIHMS1546469-supplement-2.zip › DF1/PXD009737/TestisTrypsin/TestisTrypsin_48_JARID2_VPSIIQK.pdf]

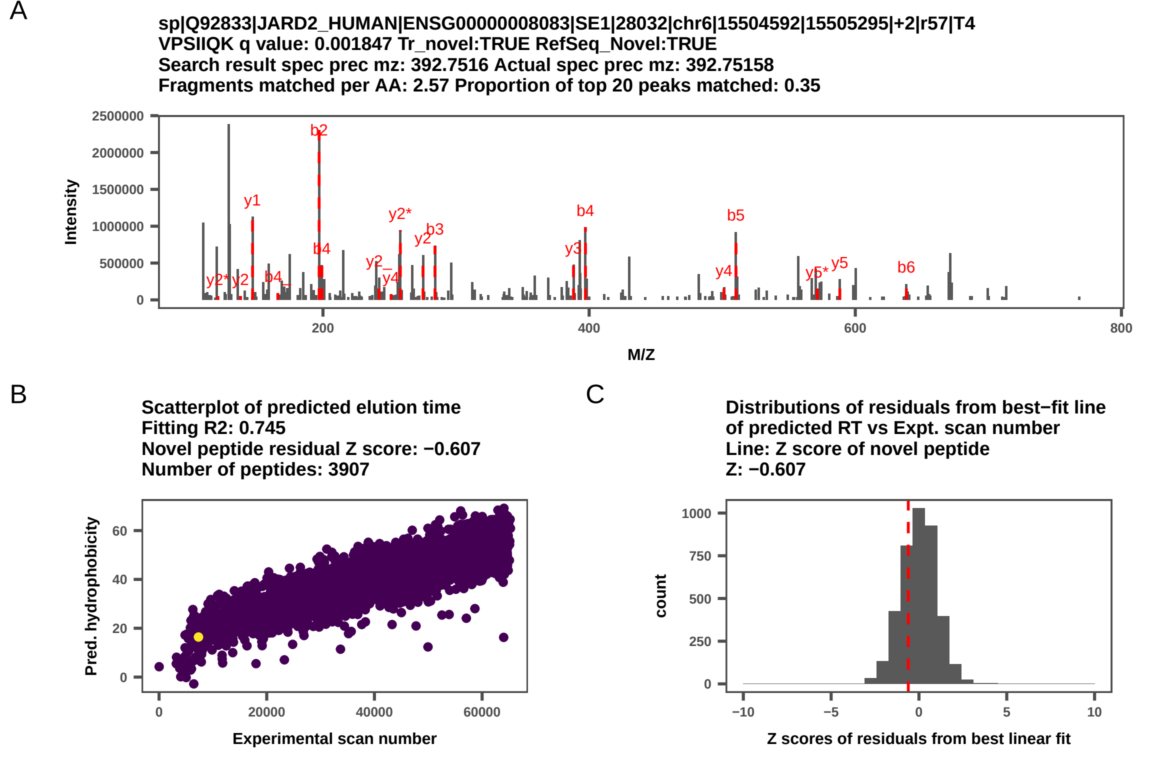
A
sp|Q92833|JARD2_HUMAN|ENSG00000008083|SE1|28032|chr6|15504592|15505295|+2|r57|T4
VPSIIQK q value: 0.001847 Tr_novel:TRUE RefSeq_Novel:TRUE
Search result spec prec mz: 392.7516 Actual spec prec mz: 392.75158
Fragments matched per AA: 2.57 Proportion of top 20 peaks matched: 0.35
Intensity
M/Z
b2
y1
y2*
b3
y2
b4
b5
y3
y4
y5
y5*
b6
B
Scatterplot of predicted elution time
Fitting R2: 0.745
Novel peptide residual Z score: −0.607
Number of peptides: 3907
Pred. hydrophobicity
Experimental scan number
C
Distributions of residuals from best−fit line
of predicted RT vs Expt. scan number
Line: Z score of novel peptide
Z: −0.607
count
Z scores of residuals from best linear fit
